# Managing severe hypertension in children

**DOI:** 10.1007/s00467-023-05896-z

**Published:** 2023-03-02

**Authors:** Malcolm G. Coulthard

**Affiliations:** Great North Children’s Hospital, Queen Victoria Road, Newcastle Upon Tyne, NE1 4LP UK

**Keywords:** Hypertension, Severe hypertension, Hypertensive encephalopathy, Children/childhood, Therapy

## Abstract

Severe childhood hypertension is uncommon and frequently not recognised and is best defined as a systolic blood pressure (SBP) above the stage 2 threshold of the 95th centile + 12 mmHg. If no signs of end-organ damage are present, this is urgent hypertension which can be managed by the slow introduction of oral or sublingual medication, but if signs are present, the child has emergency hypertension (or hypertensive encephalopathy if they include irritability, visual impairment, fits, coma, or facial palsy), and treatment must be started promptly to prevent progression to permanent neurological damage or death. However, detailed evidence from case series shows that the SBP must be lowered in a controlled manner over about 2 days by infusing short-acting intravenous hypotensive agents, with saline boluses ready in case of overshoot, unless the child had documented normotension within the last day. This is because sustained hypertension may increase pressure thresholds of cerebrovascular autoregulation which take time to reverse. A recent PICU study that suggested otherwise was significantly flawed. The target is to reduce the admission SBP by its excess, to just above the 95th centile, in three equal steps lasting about ≥ 6 h, 12 h, and finally ≥ 24 h, before introducing oral therapy. Few of the current clinical guidelines are comprehensive, and some advise reducing the SBP by a fixed percentage, which may be dangerous and has no evidence base. This review suggests criteria for future guidelines and argues that these should be evaluated by establishing prospective national or international databases.

## Introduction

Severe hypertension is rare in children, but it has the potential to cause serious harm unless it is managed with meticulous care. Historically, it was treated by the rapid reduction of the blood pressure (BP) in both adults and children, until it was recognised in the 1970s that the high incidence of ischaemic neurological symptoms in adults treated in this way [1] may be a consequence of the rise in the cerebrovascular autoregulation pressure threshold that occurs in response to severe hypertension [2], which takes time to reverse. The prediction that a slower BP reduction might allow time for the autoregulatory mechanisms to normalise was confirmed when it was shown that adults with severe hypertension had a substantially lower mortality if (a) the BP was reduced more slowly, (b) a higher initial BP target was selected, and (c) early hypotension was avoided [3]. Dillon argued that the same approach should be applied to children to prevent irreversible neurological damage in them due to rapid BP reduction causing relative hypotension and cerebral ischaemia [4, 5] and his team later demonstrated clear clinical improvement with slower BP reduction [6].

Although it has been widely accepted for decades that severe hypertension in children should be managed by the rigorously controlled slow reduction of BP, there is an important lack of consistency among existing paediatric guidelines. Furthermore, a recent report has suggested that this whole approach is flawed, stating that the ‘internationally agreed consensus … to avoid rapid BP reduction’ is ‘without clear substantiation’, and has claimed to demonstrate that ‘the risk of harm due to early and significant reduction of BP in critically ill children appears to be limited’ [7]. I wrote the present review because of this uncertainty and because of my own experience of investigating several cases of children where the late recognition and rapid reduction of severe hypertension had led to serious neurological harm and death. The purpose of this review is to (a) outline a minimum list of points that all future guidelines on managing severe childhood hypertension should clearly address and to (b) suggest a guideline to use now, based upon the existing available evidence.

## Points to be considered in childhood severe hypertension guidelines

### Which measure of BP should be used?

None of the existing published guidelines that advise on reducing severe BP in children specifies whether to focus primarily on monitoring the systolic BP (SBP) or the diastolic BP (DBP). The relative ease and precision of measuring the SBP compared to the DBP in paediatric practice [8–10], combined with the lack of normative standards for the mean arterial BP (MABP) and its lack of use outside of PICUs, support using the SBP. In addition, SBP and DBP fall in parallel when severe childhood BP is treated [11, 12], and the SBP is more sensitive than DBP at predicting the clinical severity of severe childhood hypertension [13]. I therefore suggest that future guidelines should advise monitoring the SBP as the primary target BP modality.

### What constitutes severe (stage 2) hypertension

Unlike adult BP ranges, paediatric charts are not based on proven cardiovascular risk factors, but on percentile values measured in normal childhood populations; elevated BP is defined as at least three appropriately taken values > 90th centile and hypertension when they all fall > 95th centile. However, the division of paediatric hypertension into stages 1 (mild) and 2 (severe) is based upon clinical evidence, with a substantially increased chance that end-organ damage will occur above the stage 2 threshold [14–16]. The European and US SBP-centile thresholds for stage 2 hypertension of ≥ 99th + 5 mmHg [17], and ≥ 95^th^ + 12 mmHg [18], have been shown to be equivalent [19], but I suggest adopting the ≥ 95th + 12 mmHg definition because the 95th centile values can be determined more precisely and with smaller data sets than 99th centiles [10].

Despite these definitions of stage 2 hypertension, it must be remembered that there is limited evidence that these are the ideal clinical values to decide whether a particular child requires a certain therapeutic response. For example, recent paediatric European and US guidelines suggest that an analogous BP to the adult threshold of 180/120 for a hypertensive crisis would be 20% [17] or 30 mmHg [18] above the 95th centile. There is clearly a need for clinical judgement to be applied in individual cases. It must also be recalled at all times that common factors including pain, fear and anxiety, and stress responses to intercurrent illnesses may induce transient hypertension and that measurements need to be checked by experienced clinicians to minimise the risk of acting on inaccurate BP measurements.

### Distinguishing between hypertensive urgency, emergency, and encephalopathy

Severe hypertension is divided into three clinical categories, depending on the presence of symptoms or signs, and this is an important distinction to make as it alters the approach to treatment. Urgent hypertension includes asymptomatic children, or those with non-specific symptoms of headache, fatigue, or occasionally failure to thrive [20], who can have their BP reduced gently using oral medication [17, 18]. Emergency hypertension is defined as children with evidence of end-organ damage, such as visual impairment, acute kidney failure, proteinuria, or left ventricular hypertrophy. They require their BP management to be started immediately because of the risk of sudden escalation of their condition, and to have it reduced in a tightly controlled manner using short-acting intravenous hypotensive agents [17, 18]. Hypertensive encephalopathy is when the end-organ damage includes neurological features, such as confusion, coma, fits, or a facial palsy. I suggest that these three terms are used as defined above and that adjectives such as critical, malignant, or aggressive are no longer used as labels.

### Evidence of prior hypertension

Ever since the recognition that hypertension drives an increase in the cerebrovascular autoregulatory threshold which prevents the brain tissue from being exposed to dangerously high perfusion pressures [2], it has been obvious that children who present with severe hypertension on the background of prior hypertension are physiologically very different from those who develop an acute hypertensive episode having previously been entirely normotensive. Those children whose BP was normal until hours before an acute hypertensive episode can be assumed not to have had time to have increased their cerebrovascular autoregulatory threshold and will therefore be likely to tolerate their BP being reduced back to normal promptly, whereas a rapid reduction to normal in a child with prior hypertension is likely to result in relative hypotension and cause poor cerebral perfusion [2].

Despite its obvious clinical importance, making a certain diagnosis of a child’s recent BP history is often difficult because a significant proportion of children present to hospital with severe hypertension as their first diagnosis, and no history suggesting a background cause, such as a scarred kidney which may be identified later. In addition, there is no evidence to indicate how long it takes for hypertension to cause up-regulation of the cerebrovascular autoregulatory threshold in children, so it is unclear how reassuring a moderately recent normal BP can be assumed to be. For these reasons, I suggest using two categories: those children who have had a documented normal BP measured within the last 24 h, and the group who have not, which will include cases where prior hypertension is possible, likely, or documented. Such a clear distinction is seldom made either in clinical reports or review articles that discuss managing severe childhood hypertension.

### Make appropriate age adjustments

While it is obvious that the normal range for BP varies very greatly with age, it must too be kept in mind that the clinical importance of increases in BP is also likely to be proportionate to the age of the child and to their normal upper BP limit. In practice, the significance of any particular absolute rise in BP is probably best appreciated by plotting it on an age-related chart [10]. In this review, I have plotted all of the reported childhood data as SBP values on colour-coded BP-centile ranges as defined above, with their *y* axes adjusted so their hypertension thresholds coincide regardless of age, and I graphed the adult data using the following SBP ranges: pre-hypertension ≥ 120 mmHg; stage 1 hypertension ≥ 140 mm Hg; and stage 2 hypertension ≥ 160 mm Hg [21]. The neonatal plots indicate hypertension as SBP ≥ 90th centile. I have plotted individual patient data where possible; otherwise, the mean and SD are shown.

### Clinical outcomes for patients with urgent hypertension, who were also known to have recently been normotensive

**Fig. 1:**
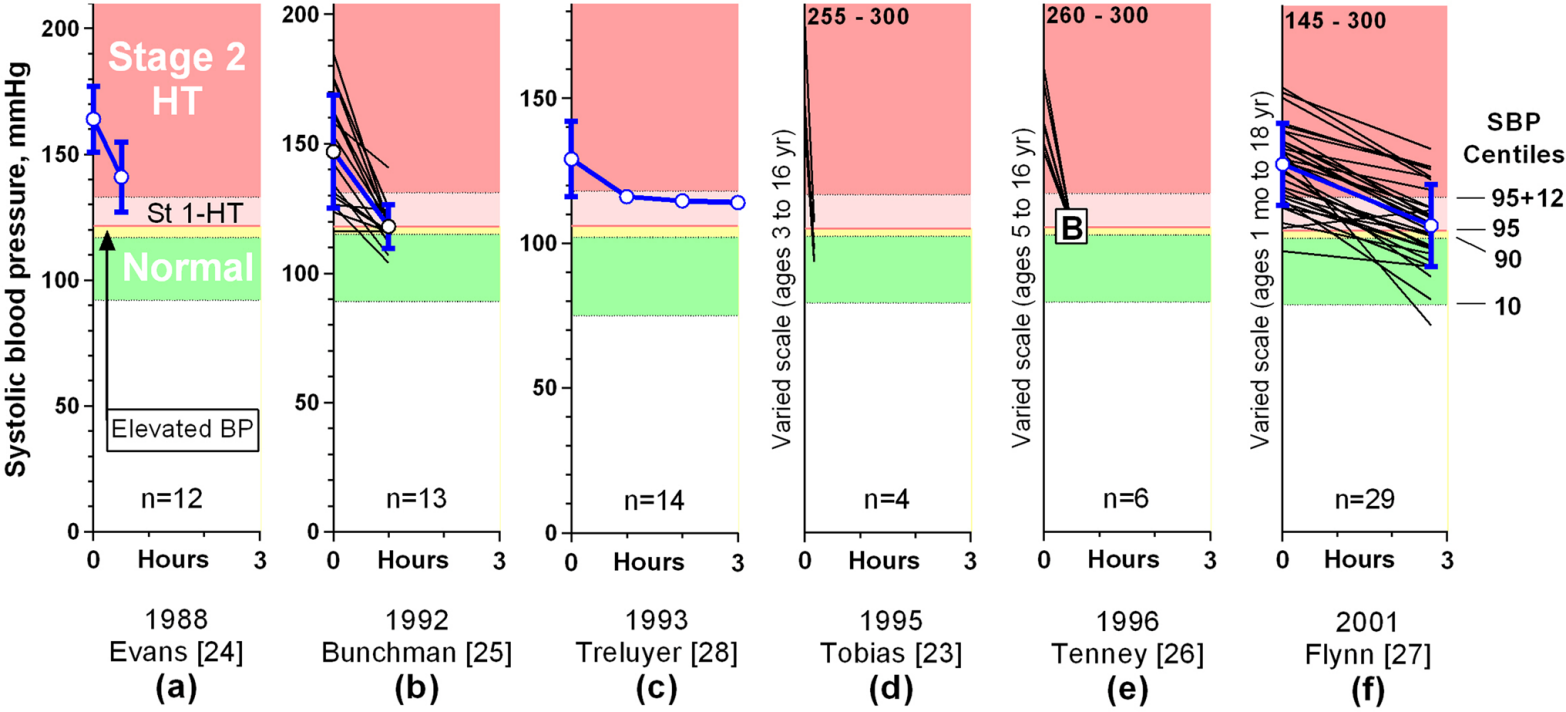
Graphs of reduction in systolic blood pressure (SBP) in 78 children from six publications, who had developed acute hypertension, having recently been normotensive. The SBP categories are labelled in graph (**a**) and the equivalent centiles are shown to the right of graph (**f**). The *y* axes have been adjusted so that the hypertension thresholds are at the same vertical height, regardless of age. Individual patient data are shown as black lines without symbols, and group data are shown in blue, with 1SD error bars where available. The letter B in graph (**e**) represents ‘their return to their BP baseline values’. None of these children developed neurological sequelae

Seven papers provide sufficient numerical or graphic data on lowering the BP in 88 children, aged from 1 month to 18 years, with urgent hypertension and documented prior normotension, to allow the rates of fall in BP to be identified, and for this to be correlated to their clinical outcomes [22–28]. All of these children had their BPs reduced rapidly to normal or near normal, and 78 cases from six reports [23–28] could be plotted (Fig. 1). The BP reduction was achieved within 15 min in two reports [22, 23], within an hour in four [24–26, 28], and within 3 h in one [27], using sublingual nifedipine [24], a rapid intravenous injection of labetalol [25], or intravenous nicardipine [22, 23, 26–28]. Individual data was provided for 46 children from three of the studies [23, 25, 27]; the SBP was reduced to < 95th centile in 25 (54%) and to < 90th centile in 17 (37%). The hypertensive episodes were triggered by being unable to take maintenance oral hypotensives (25), acute critical illnesses (23), recent organ transplants (22), renovascular disease (10), glomerulonephritis (4), and perioperative events (4). None of these children developed neurological sequelae, or died. The authors all recommended reducing the BP promptly in severe paediatric hypertension without referring to their cases all having had prior normotension, or warning that this approach may not be applicable to children presenting with pre-existing hypertension. This omission could lead some readers to incorrectly assume that it is always safe to treat severe hypertension rapidly.

### Clinical outcomes for patients with known or presumed prior hypertension

Because early hypertension may have few specific symptoms, and because children’s BP is seldom measured in primary care, many children do not present until they have severe hypertension, so prior hypertension has to be presumed. The majority have an identifiable cause, most often kidney-related, so management consists of safely reducing the BP while investigating for a pathophysiological mechanism. It is likely that increasing levels of obesity in childhood populations will increase the numbers of cases presenting with severe hypertension. Obesity is associated with a greater prevalence of essential childhood hypertension [29–31], and in one centre the mean body mass index of children presenting with severe hypertension was above the threshold for obesity [14].

**Fig. 2:**
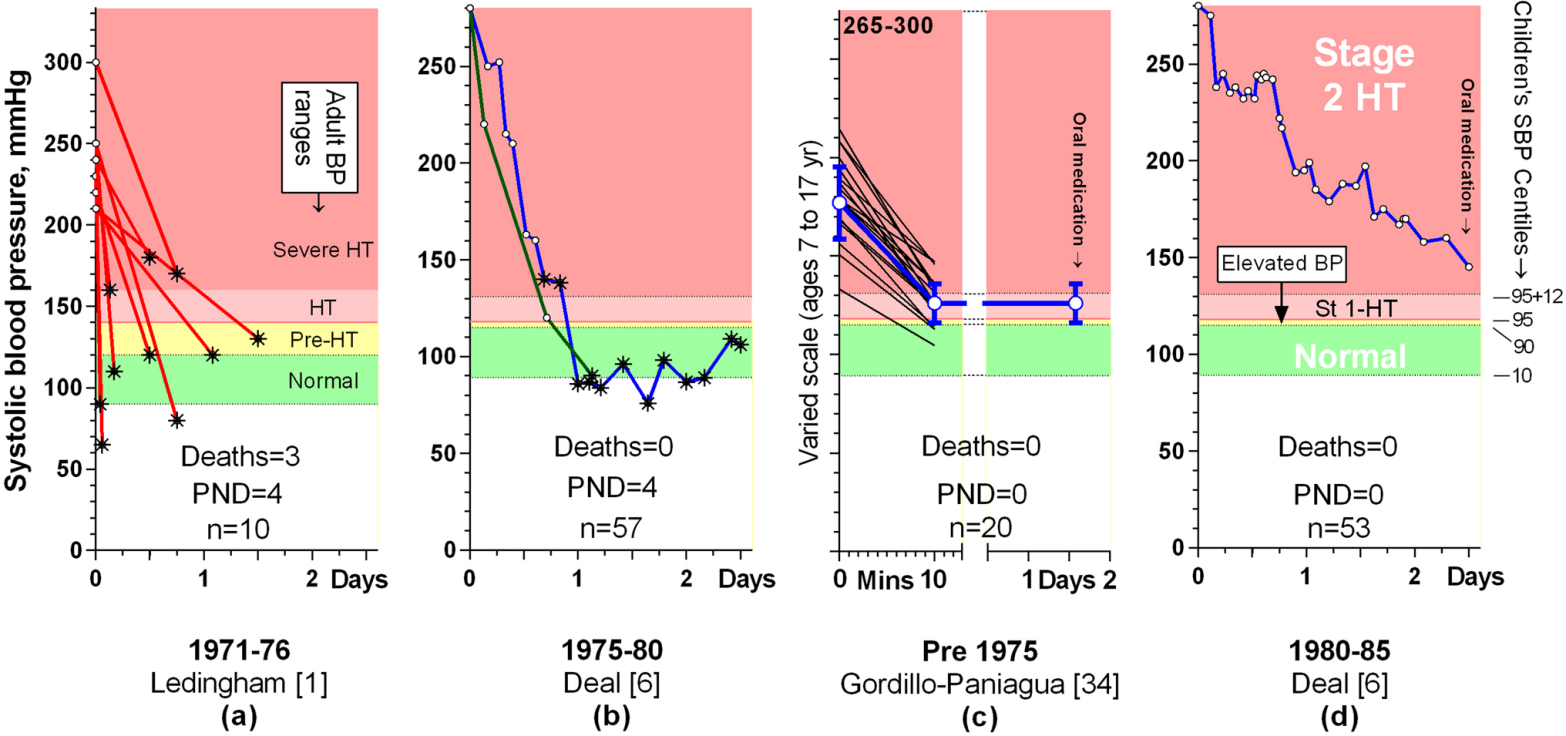
Graphs of reduction in systolic blood pressure in ten adults (**a**) and from papers that report on 130 children with severe hypertension and prior hypertension. The adult BP categories are labelled in graph (**a**), and the paediatric categories and centiles are shown in graph (**d**). The *y* axes have been adjusted so that the hypertension thresholds are at the same vertical height, regardless of age. Graphs (**a**), (**b**), and (**d**) show individual patient data, where asterisks indicate acquisition of neurological damage (the number with PND, permanent neurological damage, is also given). In plot (**c**), as in Fig. 1, black lines indicate individual patient values, with the group and 1SD in blue

The historical perspective is extremely interesting. Reducing severe adult hypertension to normal within 1 day was first shown to be associated with neurological damage or death in 1975 [1] (Fig. 2a). Dillon’s group then confirmed this to be true in children when they compared 57 children whose BP was reduced rapidly between 1975 and 1980 (Fig. 2b), with 52 children who were treated slowly during 1980–1985 (Fig. 2d) [6]. No children died in either group, but despite similar presenting hypertension profiles, 13/57 vs. 2/52 developed neurological signs or acute kidney failure during rapid treatment, and 4/57 vs. 0/52 had permanent visual loss (and paraplegia in one of them) (*p* = 0.05 for both, Fisher’s exact test). The data for the plots from Deal et al.’s paper are provided in other publications [5, 32, 33].

Also in 1975, another group reduced the SBP of 20 children with emergency hypertension or encephalitis and prior hypertension extremely rapidly (within 10 min) without inducing neurological sequelae, though one child died from a brain haemorrhage, likely caused by the hypertension itself [34] (Fig. 2c). However, the initial BP targets were mostly well above normal and sustained for long periods; 80% were in the hypertensive range (half stage 1, and half stage 2), and only the children with the mildest initial hypertension had their BP reduced to normal.

**Fig. 3:**
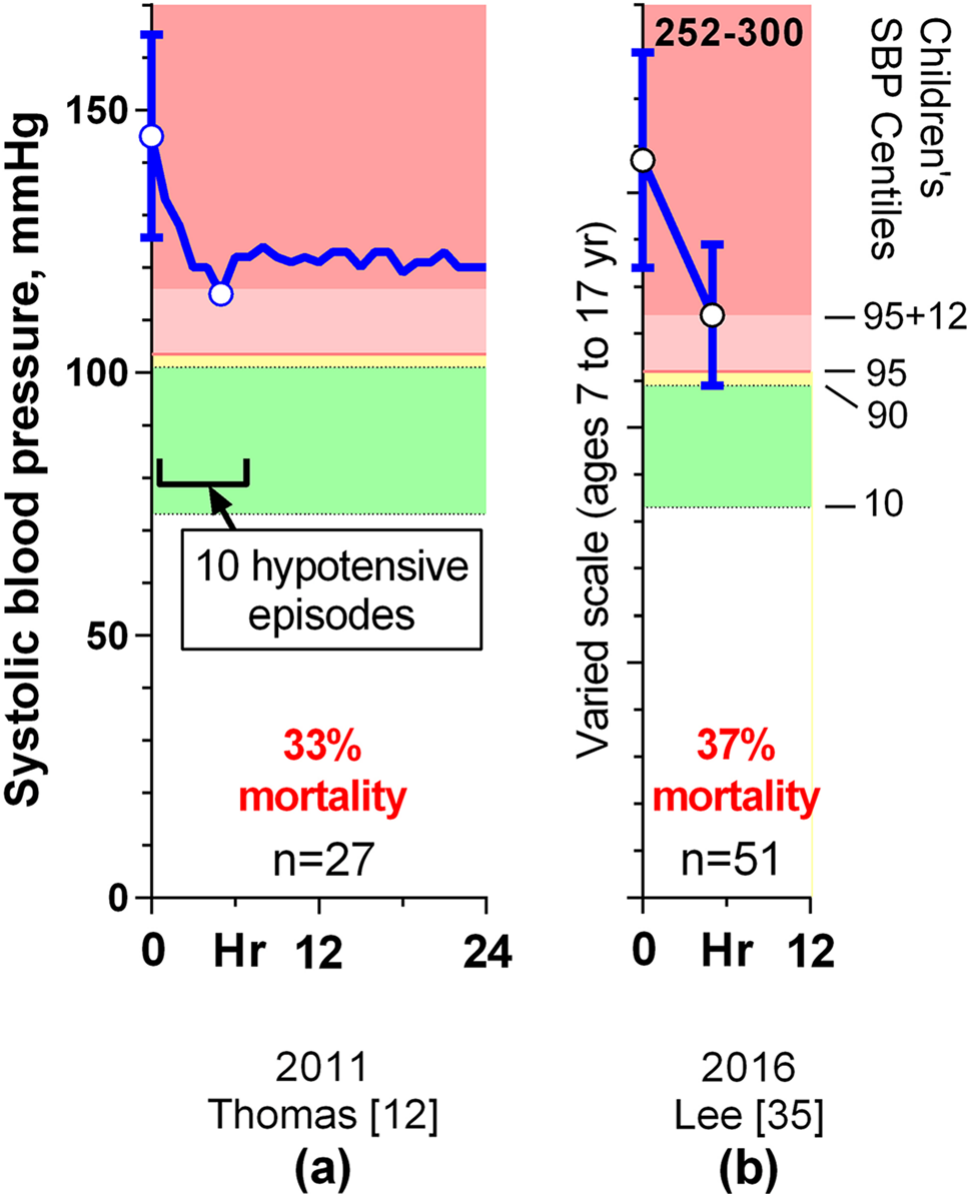
Graphs of mean reduction in systolic blood pressure in 78 children from 2 publications, with 1SD error bars. The *y* axes have been adjusted so that the hypertension thresholds are at the same vertical height, regardless of age. Mortality rates are shown

More recently, Yang reported no neurological sequelae or deaths among 55 children aged 1–18 years who presented with prior and severe hypertension (mean SBP 31 mmHg above the stage 2 threshold) and emergency hypertension or encephalopathy, and who had their BPs reduced gradually over 2–3 days [14], but there was insufficient detail to allow this to be plotted. By contrast, 28/78 (36%) of children with prior hypertension died when their severe hypertension was reduced rapidly, as detailed in two studies [12, 35] (Fig. 3). Neither of these groups mentioned mortality in their abstracts.

**Fig. 4:**
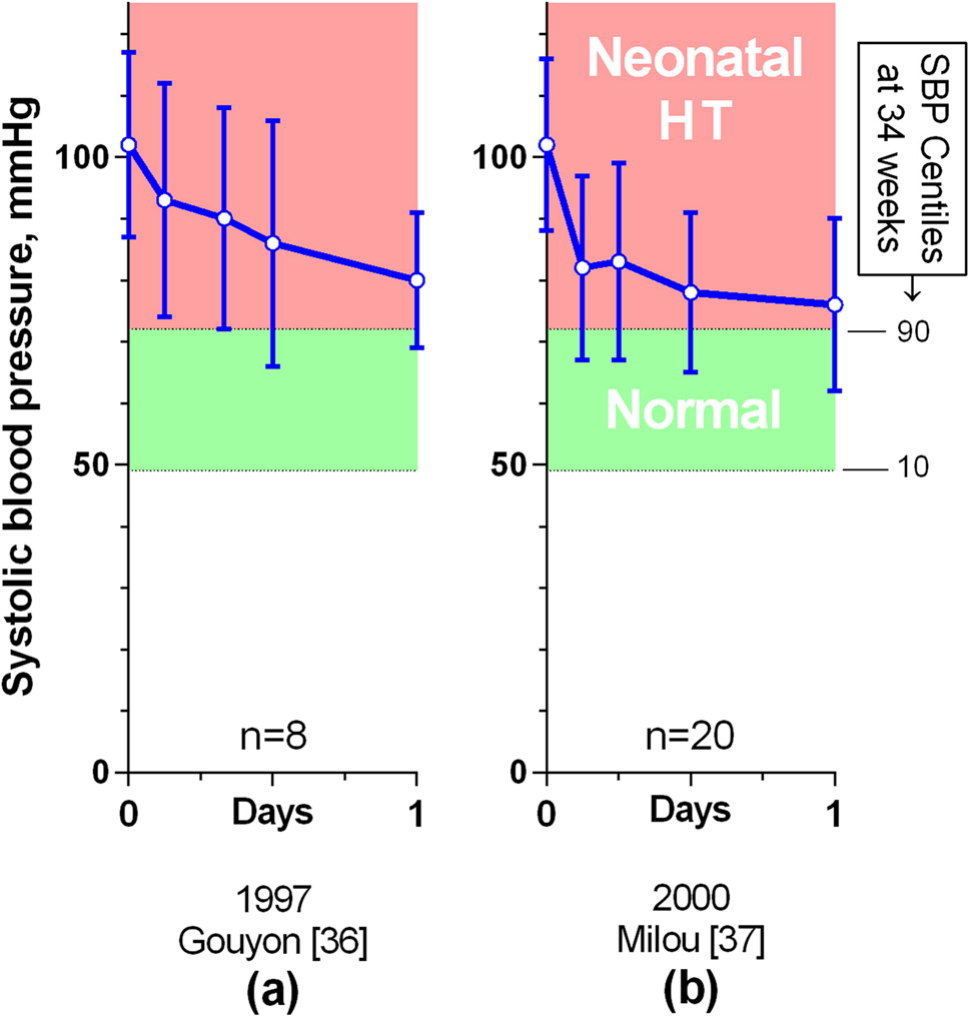
Graphs of mean reduction in systolic blood pressure in 28 preterm and new-born term infants from 2 publications, with 1SD error bars. Note that the categories of elevated BP and stages 1 and 2 hypertension have not been defined for these groups

In summary, there is good evidence that in children with severe hypertension and possible prior hypertension, a rapid reduction of the BP toward normal is dangerous, carrying a significantly increased risk of neurological damage or death, and that a controlled reduction with an intravenous infusion of a hypotensive agent over two to 3 days (which may include an early relatively brisk partial reduction) appears to be safer. Two papers from one centre that successfully treated 28 preterm and new-born term babies in this way indicate that it is also safe for that population [36, 37] (Fig. 4).

## How can a safe, slow reduction in severe hypertension be achieved?

### (a) SBP monitoring

Ideally, intensive monitoring of the SBP should be by continuous measurement via an arterial line, but until that is available, it should be done manually at least every 5 min as drugs are started or dosages changed, and at least quarter-hourly otherwise if the BP has become stable [6]. I recommend detecting the SBP with a Doppler vascular probe and inflating the cuff manually until the arterial pulse disappears and then deflating it carefully until it reappears [10]; this typically gives a clear result without the need to over-inflate the cuff which occurs with many automated devices and causes distress.

### (b) Which drugs to use?

Only continuous intravenous infusions of hypotensive agents should be used whenever possible until a pre-determined SBP target has been reached for a sufficient period, after which low doses of oral medication may be introduced. Using oral or sublingual medication early risks a prolonged period of relative hypotension, but may have a role to play if intravenous medication cannot be accessed for several hours. There is a range of intravenous agents available, but there are no well-controlled head-to-head comparative studies. Nitroprusside has a rapid response time and is awkward to use as it is light-sensitive, and prolonged use may cause cyanide toxicity. Labetalol and nicardipine are both effective, though nicardipine requires a central line to avoid thrombophlebitis [26]. In each case, commence with a low infusion rate, and then titrate the dose according to the SBP response.

### (c) Responding to relative hypotension

If the SBP falls too sharply, and crosses below the pre-set value for relative hypotension designated for that point in time, then the hypotensive drug must be stopped at once. If this does not correct the problem, it is also vital to immediately counteract the fall with rapid boluses of 5 to 10 ml/kg saline. My experience includes a 10-year-old girl reporting acute blindness while her SBP was being gently reduced, and her vision being restored immediately after a saline bolus. This advice is only provided in guidelines by Dillon’s group [4–6, 38–40] and one other author [41].

### (d) The first SBP reduction step

**Fig. 5:**
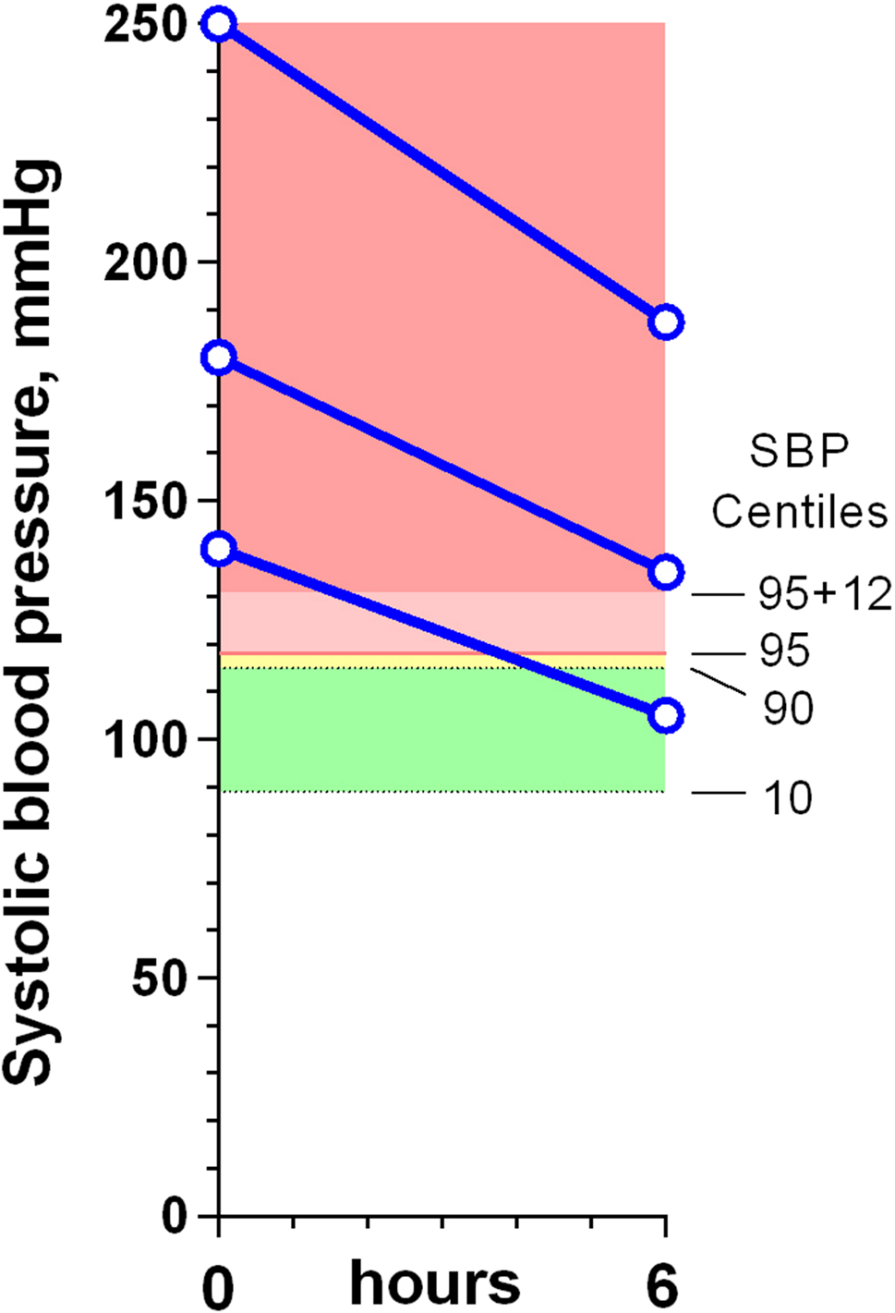
Graph showing the results of reducing the absolute SBP of an 11-year-old hypertensive child by 25%, according to the severity of their hypertension

A critical difference between published guidelines is whether the aim of the first blood pressure reduction step should be to lower it by a *fraction of the excess SBP*, such as a quarter [17, 18, 42, 43] or a third [5, 38–41, 44, 45] of the *planned reduction* required to normalise the BP, or to drop it by a *fraction of the absolute SBP*, such as by *25%* [46–50] *or 30%* of the admission value [51]. There is no paediatric evidence to support reducing the admission BP by a percentage; the first review to suggest doing this was the Fourth Task Force report in 2004 [50], which referenced Vaughan’s adult publication for justification [52], and which has been repeatedly quoted. However, Vaughan did not state this. His guidance only advised to reduce the SBP in adults with emergency hypertension by a *maximum of* 25% *so long as that target was still in the hypertensive range* (which they defined as a diastolic BP above 100 mmHg)—in other words, he specifically instructed clinicians to avoid dropping the blood pressure to normal [52]. The Fourth Task Force paper missed this vital point [50]. Figure 5 illustrates the dangers of reducing the BP by a fixed percentage; some children will remain hypertensive and others will be brought into the normal range, depending on the severity of their hypertension.

### (e) Reaching the final SBP target, before commencing oral treatment

Having defined that the only safe approach in this setting is to reduce the SBP by *its excess* above a target level over an appropriately long time before introducing oral medication, it is essential to select that target level. A value of ‘around the 95th centile’ or ‘above the normal range’ is supported by the evidence and favoured in nine reviews [4, 5, 18, 38, 39, 41–43, 53], with ‘in the normal range’ (which I interpret as < 90th centile) being suggested by one [50]. Nine authors did not define a target [17, 40, 46–49, 51, 44, 45].

There is a potential conflict between reducing the blood pressure promptly to minimise the brain’s exposure to damagingly high perfusion pressures, and not reaching normal values until the upregulated cerebral arteriolar tone has reduced. A balance is best achieved by reducing the excess SBP in three or four equally steep steps, with the initial ones being shorter. The minimum interval advised before introducing oral medication ranges from 20 h [18, 42] to 30 [17, 43, 48, 51], 34 [47, 49, 50], 40 [44], 48 [5, 38–40], and up to 78 [54] hours, with other authors simply advising to ‘do it slowly’ [4, 41, 46, 53]. One outlying author advised without evidence using oral medication to treat emergency hypertension, with the aim of achieving a 30% absolute BP reduction by 1.5 h and a normal BP within 1 day [55].

**Fig. 6:**
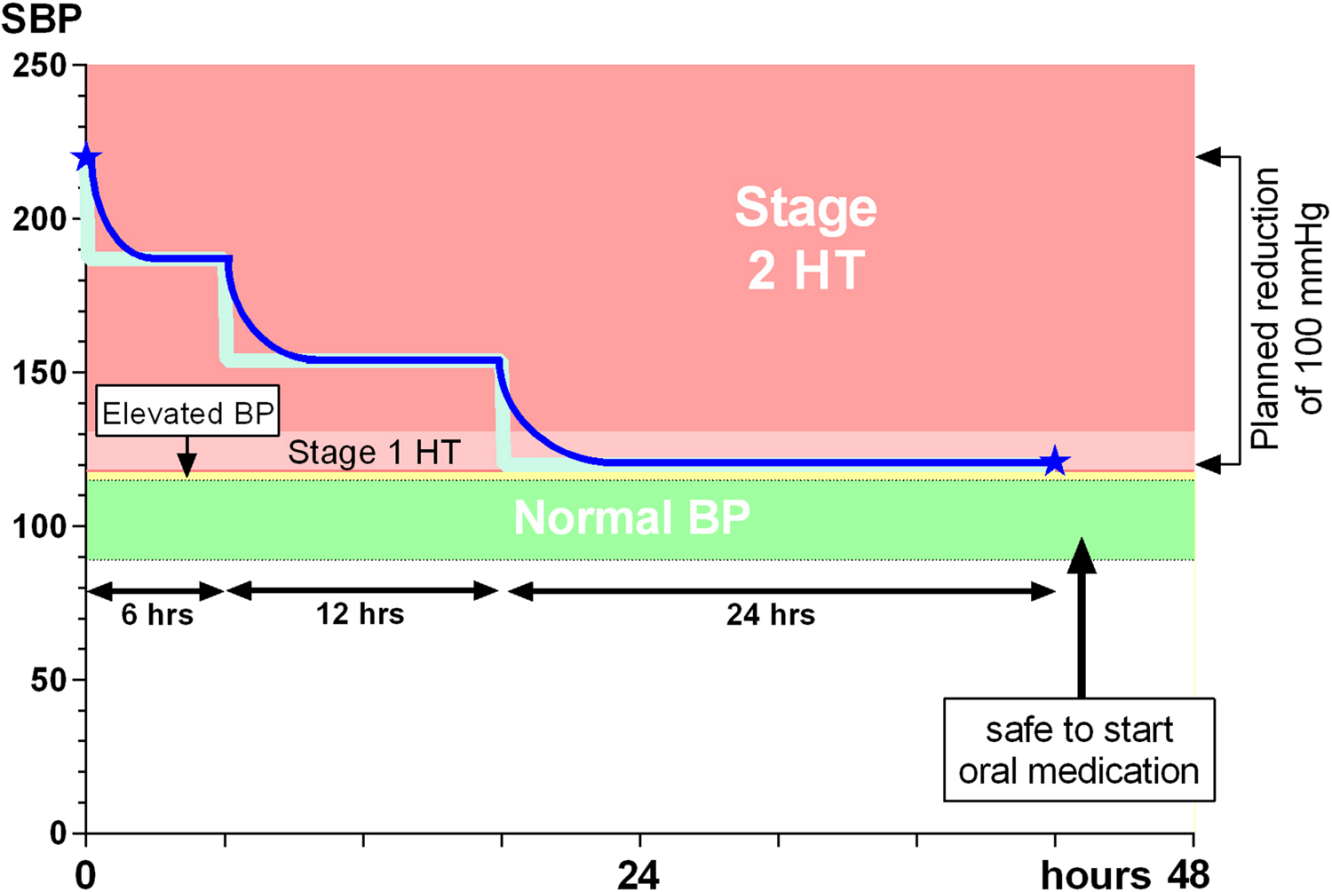
A graph to indicate a suggested guideline in a child with severe hypertension and assumed pre-existing hypertension. Thick light blue line = stepwise reduction target. Thin dark blue line = likely actual SBP path

The shortest time periods justified by the clinical evidence, and which also reflects the best consensus, is to reduce the SBP by its excess above the 95th centile target in three equal-sized steps, lasting ≥ 6 h, ≥ 12 h, and around ≥ 24 h, respectively. Figure 6 illustrates this guideline, with the step-change points shown in light blue, and the likely actual SBP trace shown in dark blue, but it is important to remember that these are minimum advisory times which should be lengthened if there is any clinical evidence of relative hypotension. To avoid confusion, it is wise to document the whole plan in advance.

## Review of an outlying study from a PICU

The authors of a PICU retrospective analysis claimed to have shown that ‘the risk of harm due to early and significant reduction of BP in critically ill children appears to be limited’ [7]. However, their study had several important flaws. First, only a minority of children they enrolled are likely to have had severe hypertension, because (a) they assessed them on a single (rather than three) SBP value and (b) their SBP threshold included all the stage 1 (mild) cases. Second, they defined a significant fall as ≤ 25% of the absolute value, which means some children will have remained hypertensive (depending on their starting value). Third, and most important, they selected an irrelevant primary outcome measure. Instead of measuring neurological damage (especially to the visual pathway), they selected without explanation the number of organ support-free days over the next month. I am unaware of any published evidence or physiological rationale to justify this. They also used mortality as a secondary outcome measure without providing a power calculation, despite the likelihood that their study design was insensitive; only 0.8% of cases were reported to have prior hypertension, and background PICU mortality rates are typical relatively high. In summary, this study did not provide convincing evidence to support reducing severe hypertension rapidly.

## An illustrative clinical case

**Fig. 7:**
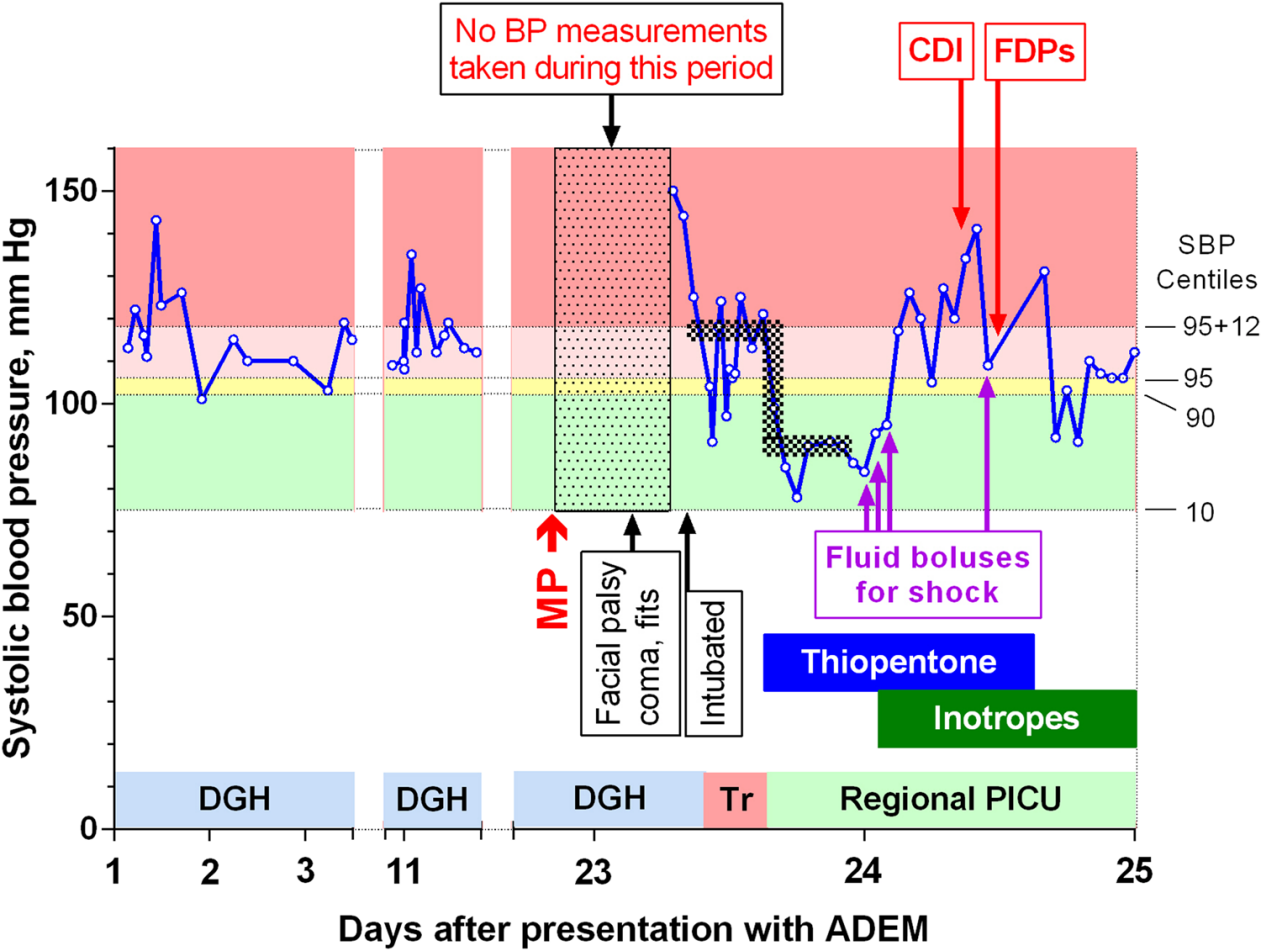
An illustrative clinical case. The lightly stippled box indicates the period without any BP monitoring, and the dark stippling shows the approximately 25% reduction in SBP after it had returned to its pre-emergency hypertension levels. CDI, central diabetes insipidus; FDPs = bilaterally fixed and dilated pupils; MP, bolus administration of methylprednisolone over 30 min; DGH, district general hospital; Tr, managed by specialist paediatric transport team; PICU, paediatric intensive care unit

In the course of providing support to families, I have recently reviewed the notes of young children rendered profoundly brain damaged due to too-rapid reduction in BP. The last case highlights some of the clinical challenges seen in managing hypertension in small children and is illustrated in Fig. 7 with her parents’ permission. A girl of 19 months developed a sixth-nerve palsy, a wide-based gait, and irritability following an upper respiratory infection and was diagnosed with acute demyelinating encephalomyelitis (ADEM) after a typical magnetic resonance scan and a raised cerebrospinal fluid protein concentration. During the diagnostic process, she had unrecognised stage 2 hypertension, a known risk of ADEM. Regional paediatric neurology advised the local hospital to administer 300 mg/m^2^ of intravenous methylprednisolone in a day-case setting, which they did without BP monitoring. Within hours she developed irritability, a facial palsy, coma, and fits, requiring multiple anticonvulsant medications and intubation, and a diagnosis of a steroid-induced hypertensive encephalopathy was made by the paediatrician and the emergency transfer team who reduced her SBP back to its previous levels, while they delivered her to the regional PICU. There, thiopentone was administered to control her fits and to rapidly reduce her admission BP by about 25% to a planned target of the 50th centile for her age (dark stippled line in Fig. 7). After being rendered normotensive for 11 h, she required fluid boluses and inotropic support for shock. During this time, she developed central diabetes insipidus and fixed pupils, which persisted until brain stem death was confirmed, and life support was withdrawn.

This case emphasises the importance of improving recognition of hypertension in small children, and avoiding risks such as hypertensive spikes from methylprednisolone infusions, either by delaying that therapy until the BP is carefully controlled or by administering it in regional centres with PICU support. It also illustrates the need for guideline clarity only to reduce the BP by *a fraction of the excess* to prevent neurological harm.

## Conclusion

There is strong evidence that childhood emergency hypertension can only be reduced promptly to normal (or its previous level) if the blood pressure has been documented to be normal within the last 24 h. Otherwise, it must be assumed that the child’s cerebrovascular autoregulation has been ‘upregulated’ and that the BP must be reduced with extreme care, using a clear plan over about 2 days, before introducing oral or sublingual agents. Many clinical guidelines are not fully comprehensive, and future ones must include all of the following components: definitions of urgent and emergency hypertension, linked to an appropriate age-related BP chart; a reminder to check prior BP measurements; clear instructions on determining the target BP and the *excess to be reduced*; a stepwise SBP reduction plan with recommended minimum timings for each phase, instruction on using a short-acting intravenous hypotensive agent and close monitoring to titrate the SBP; and a reminder to be prepared to administer saline promptly in the event of overshoot. The guidance proposed in Box 1 has all of these features.

Box 1 Guidance on managing severe hypertension in children.
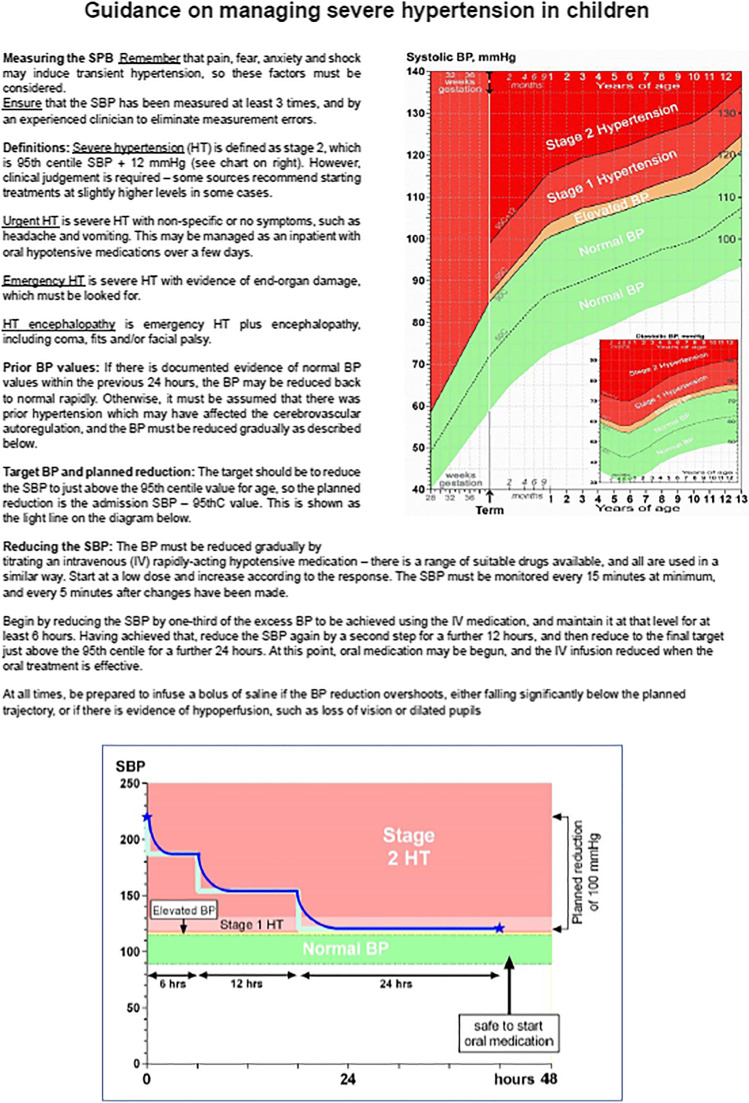


Progress in the understanding and management of uncommon but urgent childhood conditions, such as severe hypertension, inevitably come from relatively small but detailed case series generated by paediatricians with a particular interest, which means that only a small proportion of the overall cases treated contribute to our knowledge base. What is required in the future is not just the adoption of national or international guidelines which address all of the points listed above, but also the generation of co-ordinated prospective databases for all paediatricians to report to, designed to answer specific research objectives raised by those guidelines.
